# Applications of organocatalysed visible-light photoredox reactions for medicinal chemistry

**DOI:** 10.3762/bjoc.14.179

**Published:** 2018-08-03

**Authors:** Michael K Bogdos, Emmanuel Pinard, John A Murphy

**Affiliations:** 1 Department of Pure and Applied Chemistry, University of Strathclyde, 295 Cathedral Street, Glasgow G1 1 XL, United Kingdom; 2 F. Hoffman-La Roche Ltd., pRED, Pharma Research & Early Development, Roche Innovation Center Basel, Grenzacherstrasse 124, 4070 Basel, Switzerland

**Keywords:** C–H functionalisation, heterocycles, late-stage functionalisation, medicinal chemistry, organic dyes, organic photocatalysts, peptide chemistry, photoredox catalysis

## Abstract

The focus of this review is to provide an overview of the field of organocatalysed photoredox chemistry relevant to synthetic medicinal chemistry. Photoredox transformations have been shown to enable key transformations that are important to the pharmaceutical industry. This type of chemistry has also demonstrated a high degree of sustainability, especially when organic dyes can be employed in place of often toxic and environmentally damaging transition metals. The sections are arranged according to the general class of the presented reactions and the value of these methods to medicinal chemistry is considered. An overview of the general characteristics of the photocatalysts as well as some electrochemical data is presented. In addition, the general reaction mechanisms for organocatalysed photoredox transformations are discussed and some individual mechanistic considerations are highlighted in the text when appropriate.

## Review

### 1 Introduction

#### 1.1 Main advantages of organocatalysed photoredox chemistry

Photoredox catalysis is an emerging field in organic synthesis and has been the subject of many reviews in recent years [1–9]. Some cover the manipulation or installation of various functional groups [10–17], the synthesis of particular bonds (C–C or C–N etc*.*) [18–21] or the synthesis of natural products or heterocycles [22–27]. Others provide an overview of catalysts and the transformations they enable [28–33]. The most relevant review that links photoredox synthesis and medicinal chemistry is that of Stephenson [34].

Organocatalysis in general offers several advantages over transition metal-mediated catalysis. For example, removal of the catalyst during purification is much more straightforward. Furthermore, the organic molecules employed are typically much less environmentally damaging and toxic to various life forms.

Photochemistry also offers benefits compared to conventional thermally driven processes. Several transformation processes that utilise sunlight as the energy source to drive a particular reaction have been reported and some reactions presented in this review achieve this as well. This is the most energetically sustainable way possible to carry out a chemical transformation. A result of this use of the energy of photons is that photochemical transformations often require fewer and/or less reactive (which correlates to toxicity and environmental impact) components than traditional reactions.

Organocatalysed photoredox catalysis combines the advantages of both these fields. Thus, it is not only a new field filled with exciting discoveries, but also is sustainable and beneficial in the long term.

#### 1.2 General characteristics of photocatalysts

**Figure 1:**
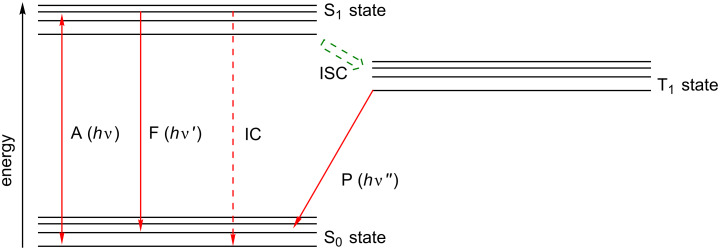
Depiction of the energy levels of a typical organic molecule and the photophysical processes it can undergo. A – absorption and emission, F – fluorescence, IC – internal conversion (non-radiative), ISC – intersystem crossing, P – phosphorescence.

**Figure 2:**
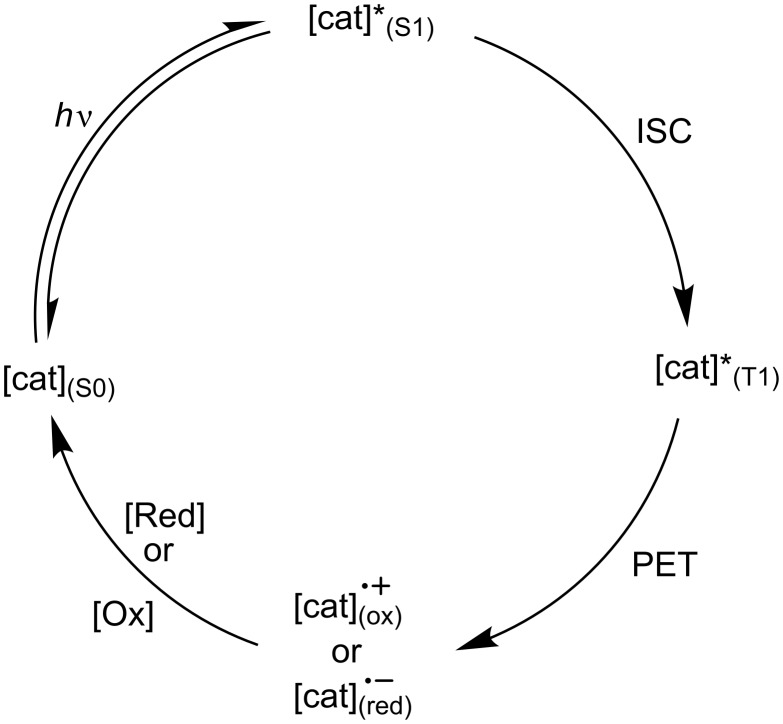
General catalytic cycle of a photocatalyst in a photoredox organocatalysed reaction. [cat] – photocatalyst, [cat]*x – photocatalyst in x (x = S_0_, S_1_, T_1_) state, ox – oxidised, red – reduced. ISC does not always occur.

**1.2.1 Brief photophysical overview.** There are several factors that affect the ability of an organic molecule to act as a photocatalyst. In a typical organocatalysed photoredox reaction, the photocatalyst transitions from a singlet ground state (S_0_) to a long-lived and relatively stable excited state, either a singlet excited state (S_1_) or a triplet excited state (T_1_), by absorption of a photon with energy *h*ν, which then undergoes photoinduced electron transfer (PET). Following this, the photocatalyst is reduced or oxidised accordingly, such that it returns to its ground state and native oxidation state (Figure 1 and Figure 2).

It is ideal if a photocatalyst has a local absorbance maximum (λ_max_) at a relatively long wavelength. Lower energy photons avoid exciting other reactants and prevent competing photochemistry from occurring, cf. ultraviolet light. However, the energy of the absorbed photon also determines the energy of the excited state of the catalyst. Catalysts with a λ_max_ at a longer wavelength have excited states at relatively low energy and therefore do not have very strong oxidising or reducing capabilities. A good balance is achieved by molecules which have λ_max_ in the visible region. Many organic molecules have some UV absorbance, but little or no absorbance in the visible part of the spectrum, hence excitation of other reactants is unlikely. Visible light photons are high enough in energy to produce excited states of sufficient reactivity to undergo PET.

Once a molecule is electronically excited, there are multiple pathways through which it can decay back to S_0_. The excited state can decay via non-radiative processes, such as vibrational relaxation. It can also return to S_0_ via fluorescence or non-radiative emission. While in S_1_ (or T_1_) Förster resonance electron transfer (FRET) can occur, a process through which energy is transferred between chromophores via non-radiative dipole–dipole coupling.

It is generally assumed that the non-radiative processes occur at a much slower rate than radiative processes. As such, the lifetime of S_1_ (τ_s1_) is roughly equal to the lifetime of fluorescence (τ_f_). In general, if a molecule is to participate in a reaction in the S_1_ state, its τ_f_ must be greater than 1 ns; N.B. the diffusion rate constant (*k*_diff_) is ≈1–2 × 10^10^ s^−1^.

The fluorescence quantum yield (Φ_f_) is another key parameter to consider when determining whether the S_1_ state of a molecule is likely to be involved in PET. A molecule with a low Φ_f_ will be unlikely to be found in the S_1_ state, as this state will be highly susceptible to other decay pathways in the timescale of PET.

For a molecule to undergo PET when in the T_1_ state, the intersystem crossing quantum yield (Φ_ISC_) must be comparable to or larger than the Φ_f_ and, more importantly, the rate constant for ISC (*k*_ISC_) must be similar to the rate constant for fluorescence (*k*_f_). The lifetime of the T_1_ state (τ_T1_) is generally orders of magnitude longer than the timescale of electron transfer (ET), meaning that τ_T1_ does not alter the efficiency of the PET process.

The decay of T_1_ is negligible as the processes which bring this about (phosphorescence mainly) are symmetry forbidden and hence very slow. Therefore, if a molecule can reach the T_1_ state through excitation with visible light then it is one which can be considered as a photoredox catalyst, as it will likely be able to participate in ET.

**1.2.2 Brief electrochemical overview.** In this review, the notation proposed by Nicewicz in his comprehensive review is adopted [35]. Therefore, all reduction potentials will be referred to using notation of the form *E*_x_ (Ox/Red) where x = “red” or “ox” and the species in the brackets refer to the reactant and product of the half reaction (Equation 1).

[1]
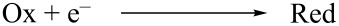


where Ox = oxidised form and Red = reduced form of the species in question.

The half reaction is always assumed to be written in the direction where reduction occurs.

[2]
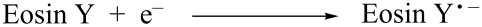


So, for example in half reaction (2) the redox potential is referred to by the notation *E*_red_ (Eosin Y/Eosin Y^•−^). The symbol “*” serves to denote when a species is in an excited electronic state, which then leads the general format adopted in this review to describe redox potentials of photocatalysts to become E^*^_x_ (Ox/Red).

The electrochemical data of a photocatalyst and a substrate which is to undergo PET allow for the estimation of the feasibility of the PET.

The following equations can be used to estimate whether PET from a substrate to an excited state photocatalyst is possible:


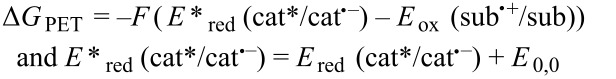


Conversely, the following equations can be used for predicting whether PET from an excited state photocatalyst to a substrate is spontaneous:


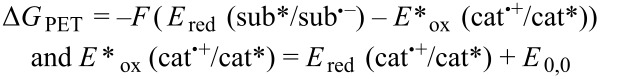


In both cases Δ*G*_PET_ is the free energy change during PET, *F* is the Faraday constant and *E*_0,0_ is the energy of the excited state.

From these equations one can conclude that for PET to take place such that the excited catalyst is reduced, E^*^_red_ (cat^*^/cat^•−^) must be greater (more positive) than *E*_ox_ (sub^•+^/sub). Conversely, for PET to occur from the excited photocatalyst (oxidation) to the substrate, E^*^_ox_ (cat^•+^/cat^*^) must be more negative than *E*_red_ (sub/sub^•−^).

Where comments or suggestions are made about the reducing or oxidising power of a photocatalyst, comparisons of relevant data and these electrochemical considerations have been undertaken.

#### 1.3 Most common catalysts employed

The following photocatalysts are frequently encountered in the literature presented in this review. Here some basic data of these photocatalysts are presented, to serve as an easy reference to the reader, with respect to their structure, electrochemical and photophysical properties.

**Figure 3:**
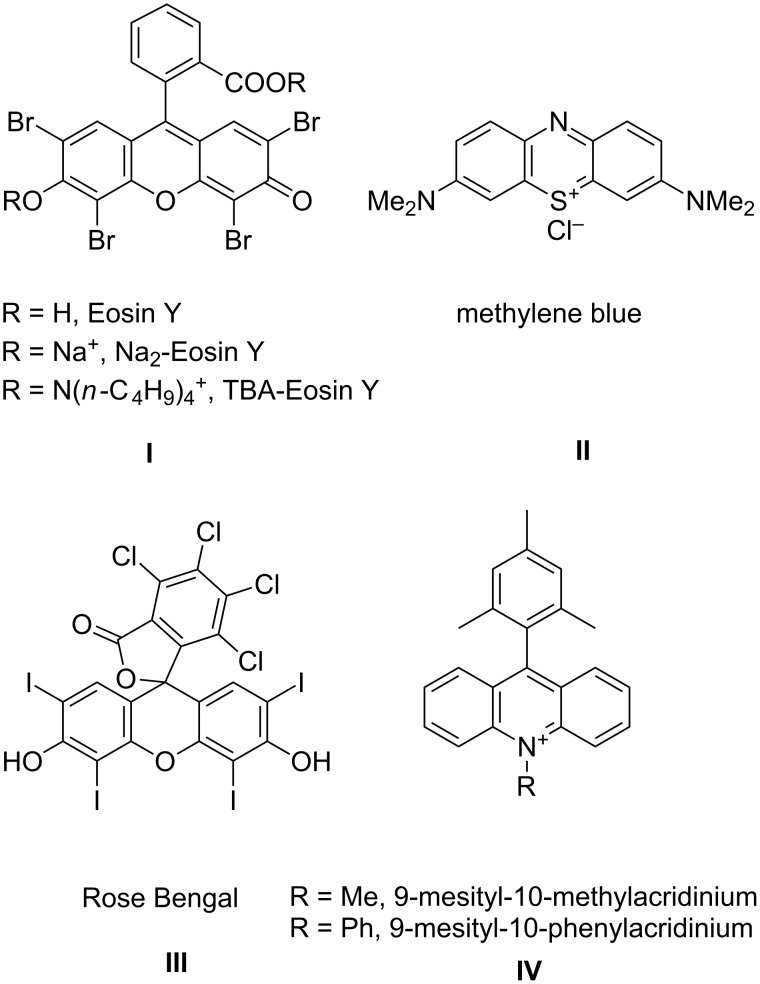
Structures and names of the most common photocatalysts encountered in the reviewed literature.

Figure 3 shows the structures of the various compounds that are used on multiple occasions as photocatalysts in the reactions presented in this review. In cases where photocatalysts other than these are used, their structure will be given in the reaction scheme.

In Table 1 the redox potentials for the ground and excited states of these catalysts are shown.

**Table 1:** Electrochemical data for the four catalysts (**I** to **IV**) most commonly encountered in the reaction reported herein [33,35–37]. All values are with respect to the saturated calomel electrode (SCE).

catalyst	ground state redox potential		excited state redox potential		excited state	absorbance maximum (λ_max_)

	*E*_red_ (cat/cat^•−^)	*E*_ox_ (cat^•+^/cat)	E^*^_red_ (cat^*^/cat^•−^)	E^*^_ox_ (cat^•+^/cat^*^)		

Eosin Y (**I**)	−1.08 V	+0.76 V	+0.83 V	−1.15 V	triplet	520 nm
methylene blue (**II**)	−0.30 V	+1.13 V	+1.14 V	−0.33 V	triplet	650 nm
Rose Bengal (**III**)	−0.99 V	+.84 V	+0.81 V	−0.96 V	triplet	549 nm
MesAcr (**IV**)	−0.46 V to −0.79 V	–	+2.32 V	–	singlet	425 nm

Examination of these data reveals how excitation of these compounds changes their properties and makes them capable of redox chemistry. In some cases, e.g., MesAcr, the produced excited state is only strongly oxidising, in others the excited state is a molecule which can both reduce and oxidise different species, depending on their relative redox potentials.

#### 1.4 General mechanism for organophotoredox-catalysed reactions

**Figure 4:**
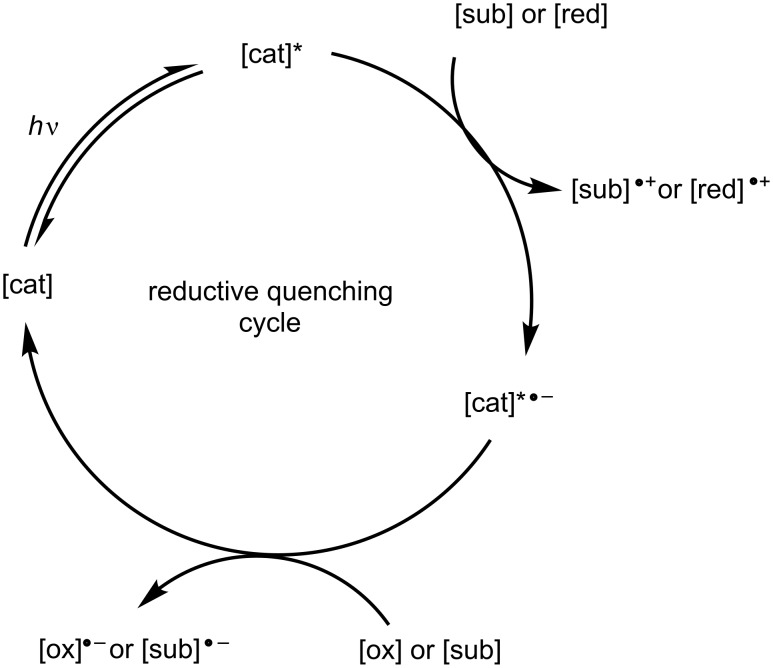
General example of a reductive quenching catalytic cycle. [cat] – photocatalyst, [cat]* – photocatalyst in excited state, [sub] – substrate, [red] – reductant, [ox] – oxidant.

The general catalytic cycle of a photocatalyst in any given organocatalysed photoredox reaction (Figure 2) can be categorised based on the direction of ET involving the excited catalyst. If ET occurs such that the catalyst is reduced, the cycle is classed as a reductive quenching cycle (Figure 4). In a reductive quenching catalytic cycle, a species must act as an oxidant to return the photocatalyst to its native oxidation state.

**Figure 5:**
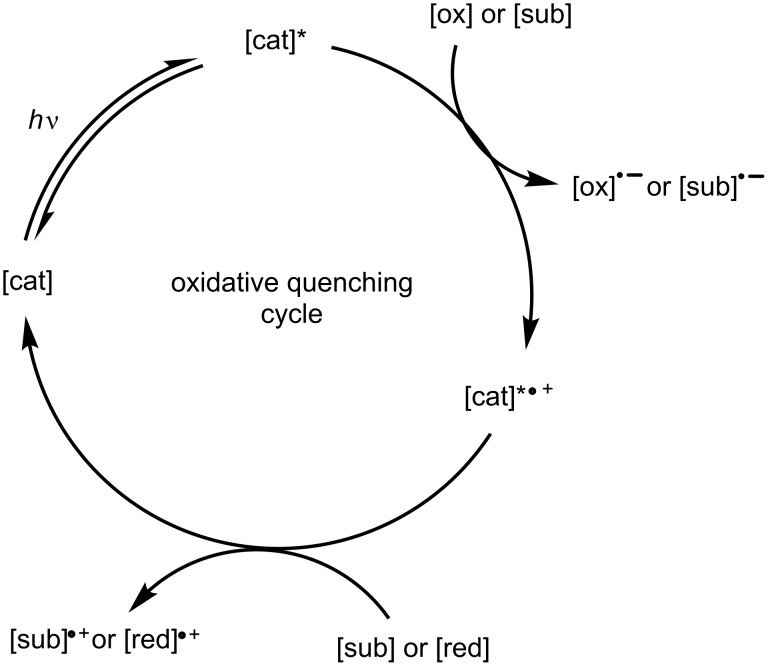
General example of an oxidative quenching catalytic cycle. [cat] – photocatalyst, [cat]* – photocatalyst in excited state, [sub] – substrate, [red] – reductant, [ox] – oxidant.

If ET occurs such that the catalyst is oxidised, the cycle is classed as an oxidative quenching cycle (Figure 5). In an oxidative quenching catalytic cycle, a species must act as a reductant to return the photocatalyst to its native oxidation state.

The reducing and oxidising species can be the substrate or an additive, since the classification of a catalytic cycle as either reductive or oxidative quenching considers only the ET with respect to the excited photocatalyst.

Photoredox reactions can also be classified with respect to the substrate. Depending on the change in oxidation state of the substrate, a photoredox reaction can be classified as either net oxidative, net reductive or redox neutral. The net result of the reaction is not dependent on the catalytic cycle characterisation – either reductive or oxidative quenching.

Net oxidative reactions require the presence of an oxidising agent. The advantage of photoredox net oxidative reactions, compared to conventional oxidation reactions, is that much milder oxidants are employed. For example, the oxidation of alcohols to carbonyls traditionally requires strong oxidants (Cr(VI) species, IBX, DMP), whereas similar reactions using photochemical methods can utilise oxygen (O_2_) as the oxidising agent. The oxidising agent can accept electrons either from the excited photocatalyst or the radical anion catalyst in the catalyst turnover step. Irrespective of its role, the reaction is still classed as net oxidative.

Similarly, net reductive transformations require a reducing agent as an additive. As with net oxidative reactions, the reductant can act at any point in the catalytic cycle, which itself can be classed either as reductively or oxidatively quenched.

Net redox neutral processes see the substrate remain at the same oxidation state overall. These transformations are generally more complex, and the additives required as well as their mode of action vary in each case.

#### 1.5 Scope, aim and selection criteria for presented publications

Unless otherwise stated, control experiments were completed to prove the necessity of the light source, photocatalyst and all additives in the reported reactions. In addition, unless otherwise stated, optimisation of the reaction conditions was carried out for all components of the reactions presented.

Only selected mechanisms are reported, as the focus is on the applications of the presented reactions in the synthesis of molecules for the purposes of medicinal chemistry. For the sake of brevity, most mechanisms are omitted completely, as they can be correctly inferred by the reagents, conditions and general mechanisms described previously (section 1.4). If the mechanism contains information vital to the understanding of various results, the key steps which contain this information will be presented and discussed.

This review aims to function as a kind of synthetic medicinal chemists’ guide to organocatalysed visible light photoredox chemistry. For this reason, the review is structured such that reactions that fall under a broad category are grouped together. The main text is separated into three sections, which correspond to reactions frequently used in medicinal chemistry.

The existing literature on the topic is extensive. The papers reviewed were selected on the following criteria:

- The reactions described must not be extremely common, easy to undertake using more traditional methods or overly simplistic, e.g., simple functional group transformations.
- The publication presents a new idea or breakthrough that can potentially significantly impact the field of medicinal chemistry or synthetic organophotoredox catalysis.
- The conditions reported offer a distinct, unique or significant advantage over non-photocatalytic or transition metal photocatalysed processes.
- The reported chemistry has no precedent in the literature and is only possible using organophotoredox chemistry.
- The products presented must always be somehow important in medicinal chemistry.
- A combination of some of the above conditions.

As such, the number of research papers reviewed and presented is by no means exhaustive but is an attempt to present the content which is most relevant.

### 2 Coupling reactions

In this section, the bonds formed during the reactions are highlighted in red, as they are not always immediately and easily identifiable.

#### 2.1 Peptide-type linkages

**Scheme 1:**
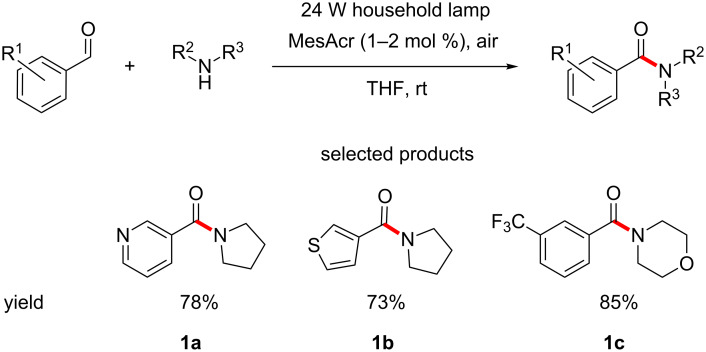
Oxidative coupling of aldehydes and amines to amides using acridinium salt photocatalysis.

Medicinal chemists often draw inspiration from nature for the design of their molecules. With the exception of certain natural products, most naturally occurring biologically active molecules contain amides. The formation of the amide functional group is the most utilised reaction in medicinal chemistry. This is a reflection of the tendency of synthetic bioactive molecules to exhibit peptidomimetic properties. Though there is a vast number of procedures that generate amide bonds, an interesting approach is taken by Leow, who has demonstrated the synthesis of amides through the oxidative coupling of aromatic aldehydes and a wide range of secondary amines, using mesitylacridinium salts as the photocatalysts (Scheme 1) [38].

The main advantage is the use of air as the oxidant, which converts the formed α-hydroxy amine into the desired amide. This makes for a much more atom economical and environmentally benign process, when compared to traditionally used amide coupling methods where acid activating agents are needed, e.g., HATU or DCC, as the only byproduct is water.

Only arylaldehydes can be converted into amides and all but one of the examples of the amines used are secondary cyclic amines. Aliphatic aldehydes gave poor yields, with sterically hindered examples not reacting at all. The author attributed this to the formation of enamines. Under the reported reaction conditions primary aliphatic and aromatic amines all produced imines.

**Figure 6:**
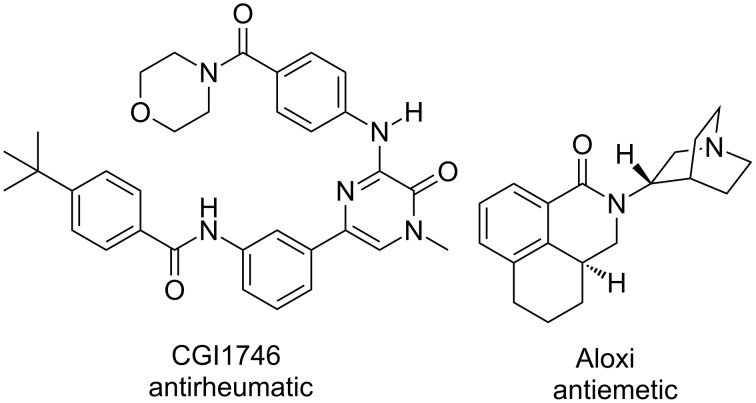
Biologically active molecules containing a benzamide linkage.

The benzamide moiety is somewhat common in biologically active molecules. Leow recognised this and provided some examples which are currently on the market or under investigation (Figure 6) [39]. An example of the formation of these bonds using his protocol would serve as a demonstration of utility of the reaction to complex, biologically relevant systems.

Naturally occurring amino acids, e.g., glycine, are often used in medicinal chemistry as linkers, structural components of scaffolds or even as precursors to useful building blocks.

**Scheme 2:**
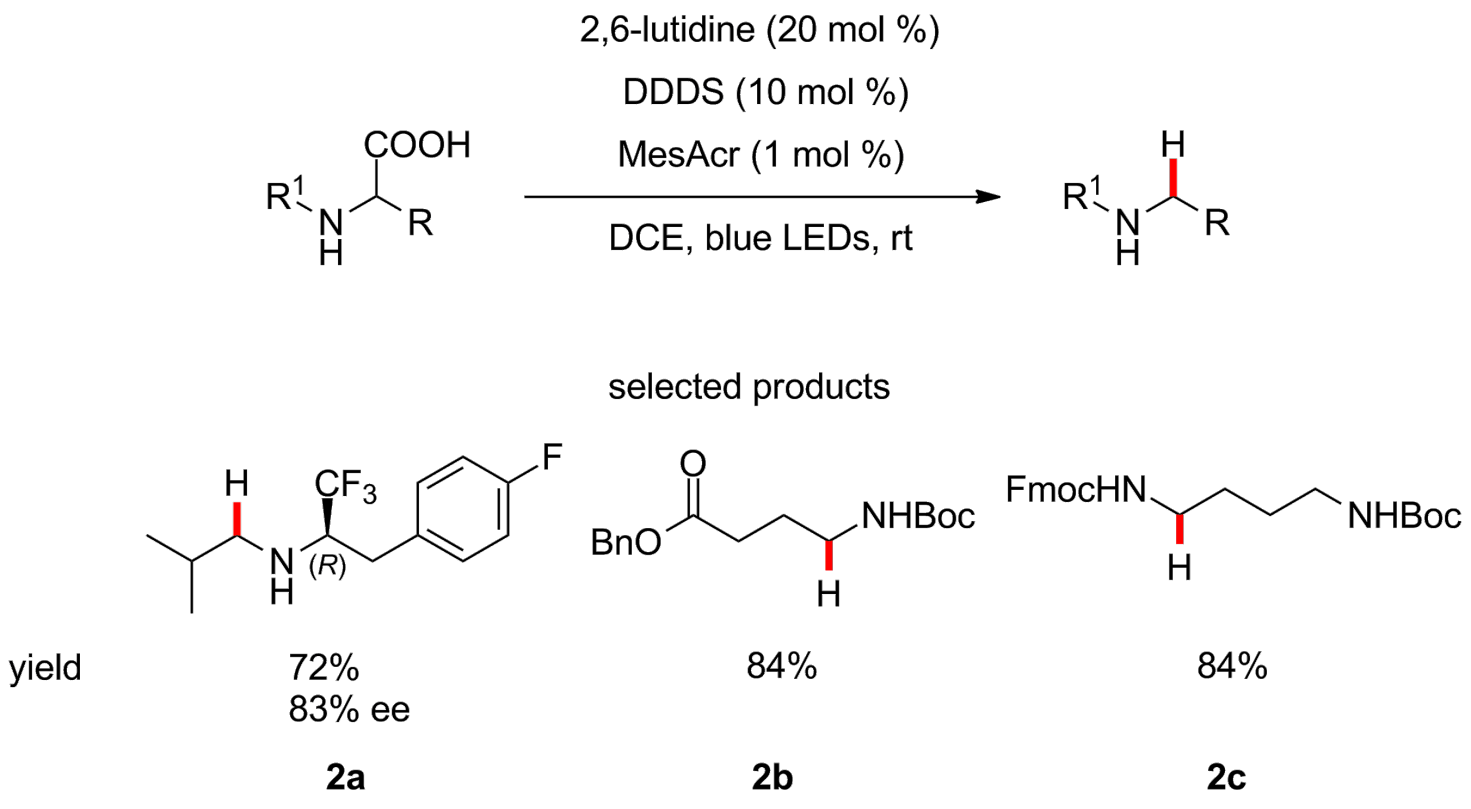
The photocatalytic reduction of amino acids to produce the corresponding free or protected amines.

Wallentin and co-workers have described a method for the reductive decarboxylation of amino acids, using bis(4-chlorophenyl)disulfide (empirical name – dichlorodiphenyl disulfide, abbreviated as DDDS), 2,6-lutidine and acridinium salts under blue LED irradiation, providing access to precious, non-commercially available and multifunctional amine building blocks in one step (Scheme 2) [40].

The group demonstrated the synthesis of protected naturally occurring amines such as GABA and phenylethylamine as well as diamines with orthogonal protecting groups, cf. product **2c**.

The combination of this protocol with the diastereoselective reductive amination reported by Hughes and Devine [41], provides access to very high value chiral α-trifluoromethylamines, which are attractive due to their low basicity, ability to act as amide bioisosteres [42] and higher metabolic stability.

The number of medicinal chemists who specialise in peptides has been increasing in recent years. This is largely due to the increasing interest in macrocyclic peptides. A key structural characteristic of peptides is the disulfide bridge formed by cysteines. This functional group is much more prevalent in peptide medicinal chemistry.

**Scheme 3:**
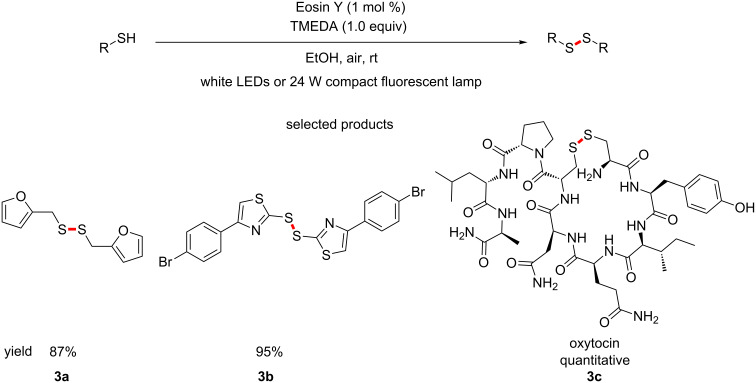
The organocatalysed photoredox base-mediated oxidation of thiols to disulfides.

Noël et al. have published a protocol for the aerobic oxidation of thiols to disulfides, using Eosin Y photocatalysis and TMEDA (Scheme 3) [43].

The reaction specifically investigated the dimerization of thiols. Some of the experiments carried out by the group were in a flow chemistry set up, exemplifying the scalability of the procedure. In addition, the oxidant that achieves the transformation is molecular oxygen, making this a very sustainable route, in a similar manner to the amide coupling by Leow.

The demonstrated oxidation of the free thiols to a disulfide to afford oxytocin (**3c**) as the product in quantitative yield shows the value of this procedure. Medicinal chemists who specialise in creating artificial peptides could find much use for such a mild and selective oxidation.

**Scheme 4:**
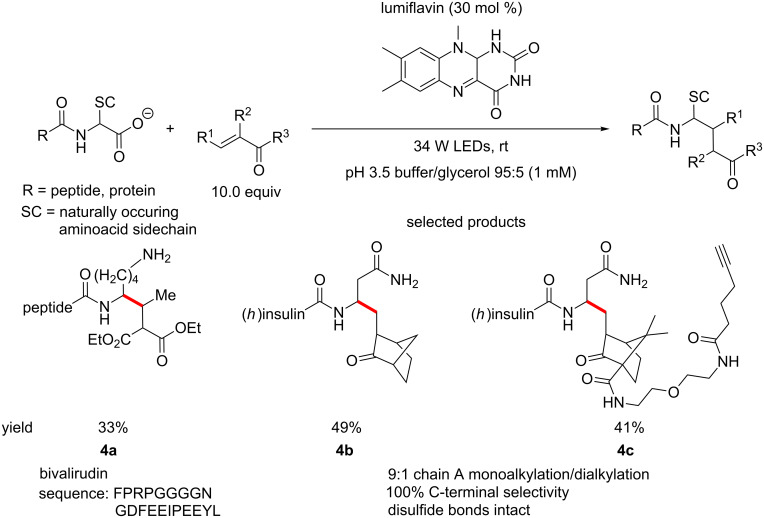
C-Terminal modification of peptides and proteins using organophotoredox catalysis.

MacMillan and co-workers have recently developed a method for the bioconjugation of peptides by radical decarboxylation of the C-terminus of peptides and subsequent Giese-type addition to Michael acceptors. This is performed under blue light irradiation, using lumiflavin (^3^Lum *E*_red_*(cat/cat^•−^) ≈ +1.5 V vs SCE) as the photocatalyst in aqueous buffer (Scheme 4) [44].

This work highlights the great biocompatibility of organophotoredox methodology and also is an excellent demonstration of the type of chemoselectivity achievable using these methods.

Apart from the immediately obvious application of this bioconjugation to biochemistry and molecular biology as a tool for protein labelling, it could also be used as a convenient tool for peptide chemists in medicinal chemistry programs for modification of peptides.

#### 2.2 C(sp^2^)–C(sp^2^) bond formation

Suzuki–Miyaura and related transition metal-catalysed C–C bond forming reactions are in the top 5 most used reactions in medicinal chemistry [45]. Therefore, the development of metal-free variants of these types of reactions is a very attractive goal.

**Scheme 5:**
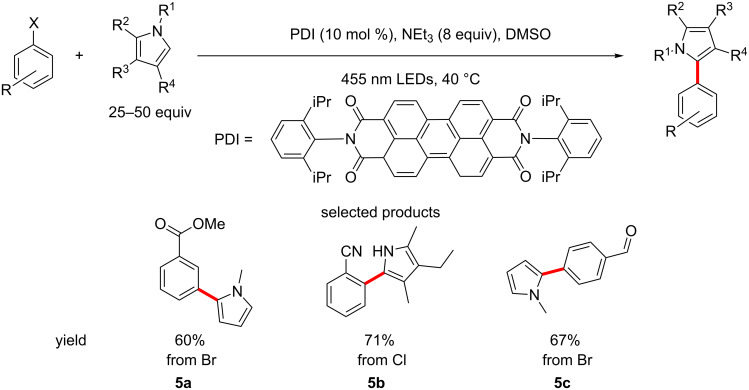
The reduction and aryl coupling of aryl halides using a doubly excited photocatalyst (PDI).

An interesting approach was taken by König et al., who report the reduction of aryl halides to the corresponding non-halogenated aromatics. This was extended to the coupling of aryl halides to a variety of substituted pyrroles, using *N,N*-bis(2,6-diisopropylphenyl)perylene-3,4,9,10-bis(dicarboximide) (PDI) as the photocatalyst, under blue LED irradiation, in DMSO and in the presence of triethylamine (Scheme 5) [46].

The biggest breakthrough in this case is the excitation of PDI by two photons, creating a radical anion in an excited state, giving the catalyst a much higher reducing power, allowing the reduction of aryl chlorides (see product **5b**). This is the first report of the reduction of aryl chlorides without the use of a strong base, UV radiation or highly reactive neutral organic reducing agents.

**Figure 7:**
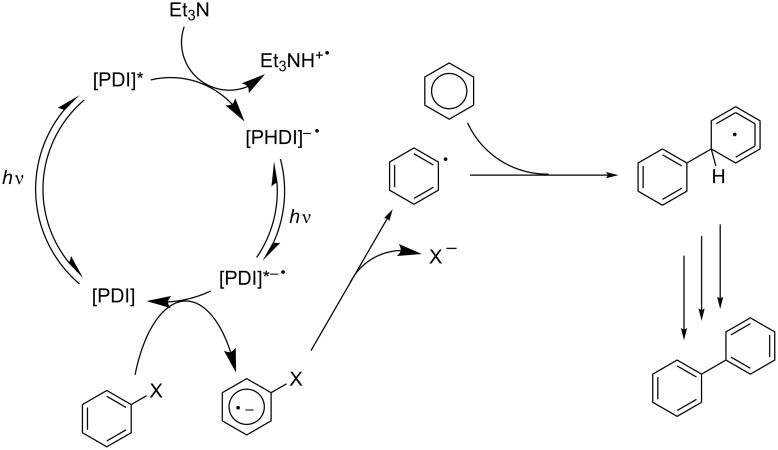
Mechanism for the coupling of aryl halides using PDI, which is excited sequentially by two photons.

The PDI catalytic cycle is different to the general catalytic cycle presented in the introduction and is presented in Figure 7.

The group also showed that it is possible to carry out these reactions using sunlight as the source of photons, leading them to term their newly discovered photocatalyst a “minimalistic chemical model of the Z-scheme in biological photosynthesis”.

**Scheme 6:**
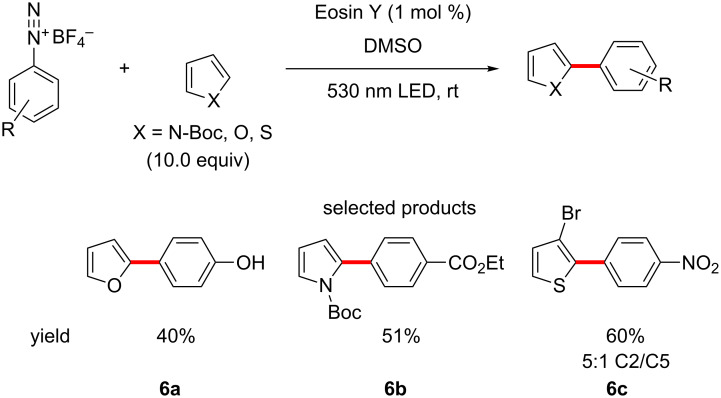
The arylation of five-membered heteroarenes using arenediazonium salts under organophotoredox conditions.

The König group has contributed a more traditional methodology as well, publishing a protocol for coupling simple five-membered heterocycles to substituted benzenes, using Eosin Y as the photocatalyst, starting from arenediazonium salts (Scheme 6) [47].

The scope of the reaction is limited to *N*-Boc-pyrroles, furans and a couple of simple substituted thiophenes with respect to the heterocycle, and the benzene moiety can tolerate all manner of substituents in all positions, however, only mono-substituted systems are explored. This is a powerful procedure, which allows for the circumvention of traditional Pd or Cu catalysed couplings of heteroarenes, which are notoriously difficult.

**Scheme 7:**
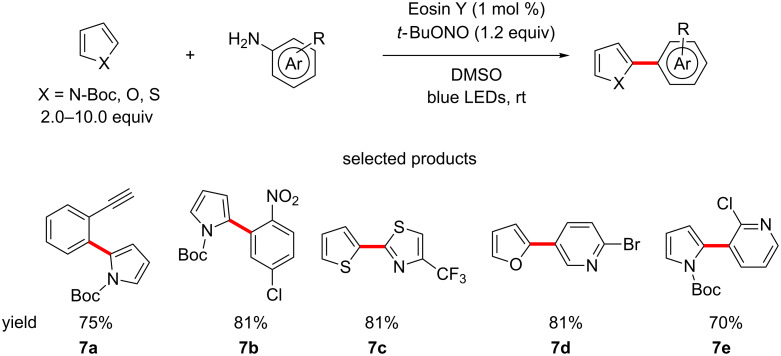
The C–H (hetero)arylation of five-membered heterocycles under Eosin Y photocatalysis.

A publication by Kundu and Ranu provides a way of arylating the C2 position of electron-rich five-membered heterocycles, using anilines as the coupling partner. *tert-*Butyl nitrite (*t*-BuONO) is used as a diazotizing agent to generate diazonium salts transiently in situ. The reaction is catalysed by Eosin Y under irradiation with blue LEDs at room temperature (Scheme 7) [48].

The authors report that in general their procedure allowed for better transformation of electron-poor anilines compared to their electron-rich counterparts. Unfortunately, it is also reported that the scope of the reaction cannot be extended to electron-poor heterocycles under the current conditions. However, the aniline coupling partner opens up a huge variety of opportunities, as arylamines and heteroarylamines are widely available.

Scaffolds such as **7b** have lots of potential for further elaboration. In addition, the presence of electron-poor pyridines, cf. **7e**, is also very encouraging, as these find widespread use in pharmaceuticals as benzene isosteres, which are more polar and metabolically stable, but lack the – often problematic – basicity of regular pyridines. Compounds such as **7c** are not commonly encountered, but definitely have the potential to be of interest if explored further. The good physical chemical properties of thiazoles, as well as their ability to act as isosteres to thiophenes, carbonyls and pyrazoles [49–50] make this scaffold an intriguing novel motif.

#### 2.3 C(sp^2^)–X bond formation

The obvious extension after considering C–C coupling reactions, is to consider the ability of organocatalysed photoredox reactions to perform reactions which create C–X (X = N, O, S) bonds, in reactions analogous to Buchwald or Chan–Lam couplings.

**Scheme 8:**
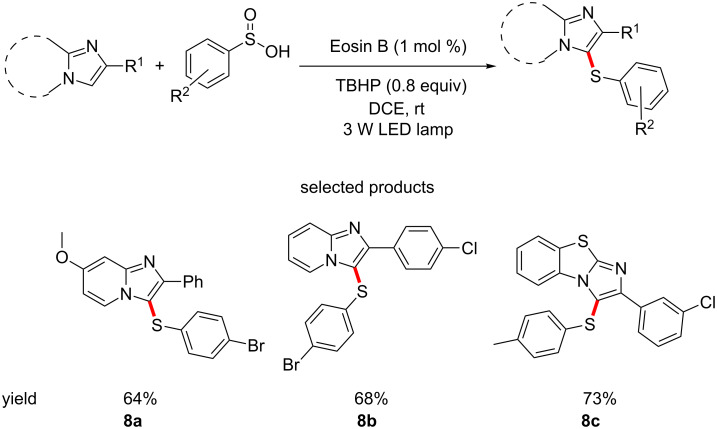
The C–H sulfurisation of imidazoheterocycles using Eosin B-catalyzed photochemical methods.

An example of the creation of C–S bonds is given by Wang and co-workers, who have reported the formation of aromatic thioethers by functionalising C–H bonds of imidazo[1,2-*α*]pyridines and benzo[*d*]imidazo[1,2-*b*]thiophenes using Eosin B and sulfinic acids (Scheme 8) [51].

The manipulated heterocycles, particularly the imidazopyridines, are motifs which are commonly encountered in medicinal chemistry. The formation of the thioether is also quite interesting, due to the possibility of accessing sulfones, which are abundant in drugs and drug-like molecules.

The scope of the reaction covers the basics in terms of substituents on both reactants and investigates various substitution patterns. Most of the reactions proceed in good yields. The use of *tert*-butyl hydroperoxide as the oxidant likely prohibits the use of oxidation-sensitive functional groups, such as alkenes or aldehydes. Use of a milder oxidant, e.g., oxygen – seen many times in this review, could help broaden the functional group compatibility.

**Scheme 9:**
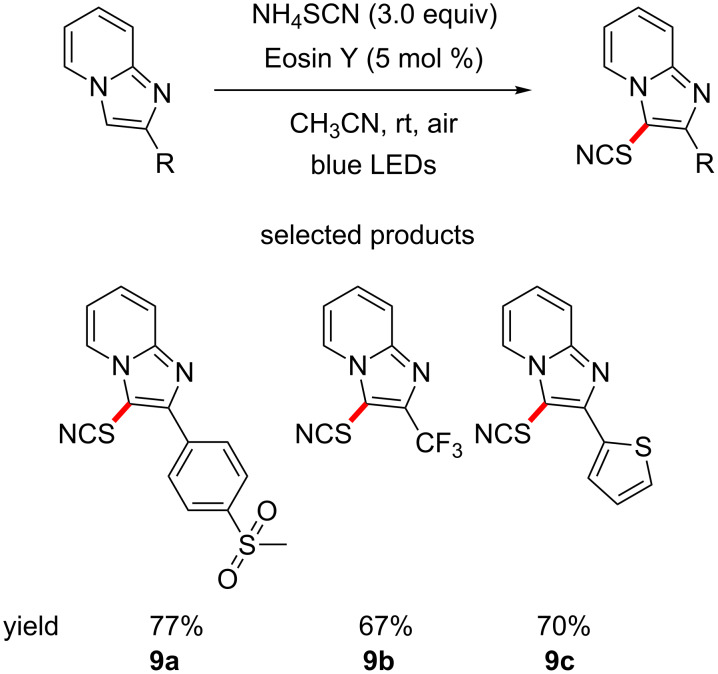
The introduction of the thiocyanate group using Eosin Y photocatalysis.

Hajra et al. have reported the direct C–H thiocyanation of substituted imidazo[1,2-*a*]pyridines, using ammonium thiocyanate, in combination with Eosin Y under irradiation by blue LEDs (Scheme 9) [52]. This is another photoredox example of C–S bond formation, in this case to a highly versatile thiocyanate functional group, which is a precursor group to many sulfur-containing functional groups, as well as heterocycles such as thiazoles and isothiazoles.

The imidazo[1,2-*a*]pyridine core is a particularly interesting drug-like structure, e.g., electron poor, polar, of low basicity, etc. The scope of the modified substituted imidazo[1,2-*a*]pyridines contains scaffolds commonly found in pharmaceuticals, such as sulfones **9a** and trifluoromethyl groups **9b**.

Hence, this publication provides an easy route to access scaffolds with diverse aromatic systems, allowing for the construction of interesting molecules.

**Scheme 10:**
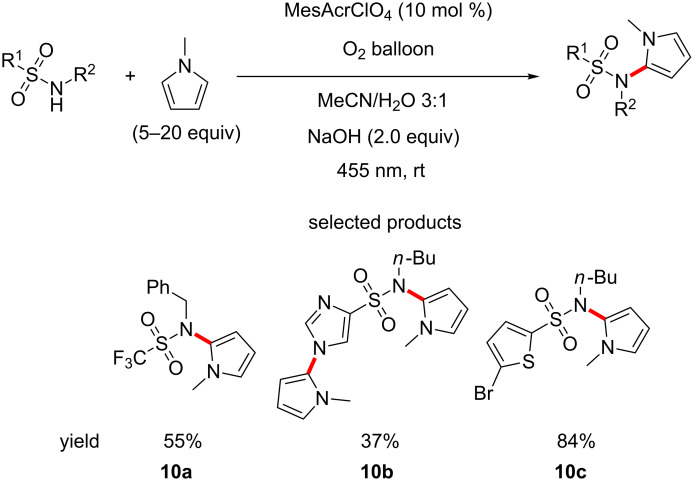
Sulfonamidation of pyrroles using oxygen as the terminal oxidant.

An interesting report of C–N bond formation is seen in König and co-workers’ method for the formation of sulfonamidated pyrroles, using acridinium salts as photocatalysts, in the presence of oxygen and sodium hydroxide (Scheme 10) [53].

Unfortunately, this protocol was investigated for its use in the sulfonamidation of other heterocycles and was not successful. The authors attribute this lack of reactivity to the limited oxidising power of the excited acridinium salts and to the relative instability of the heterocyclic radical cation, which is a key intermediate in the proposed mechanism. Considering that *E*_red_*(cat/cat^•−^) is greater than +2 V (vs SCE) for acridinium salts, the limited oxidising power is not the most probable explanation. Instability of the heteroaromatic radical cation seems more plausible. The authors explore both aromatic and heteroaromatic pendant groups on the sulfonamide, as well as aliphatic chains. Unsurprisingly, esters and other base labile groups are not encountered.

**Scheme 11:**
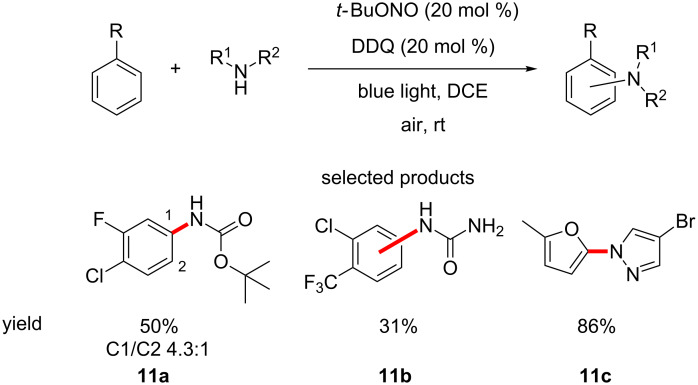
DDQ-catalysed C–H amination of arenes and heteroarenes.

A recent publication by König and his group shows the DDQ catalysed (^3^DDQ *E*_red_*(cat/cat^•−^) ≈ +3.18 V vs SCE) C–H amination of arenes and heteroarenes using weakly nucleophilic species such as carbamates, urea and non-basic heterocycles (Scheme 11) [54].

The scope covers a multitude of electron-poor and electron-rich arenes which can be reacted with carbamates, urea, pyrazole and triazole derivatives to furnish aminated products. The authors address the various reactivities observed with respect to both the electronics of the arene and the nucleophilicity of the amine. Particularly electron-rich arenes such as *N*-methylindole are not tolerated, as is the case for relatively nucleophilic amines such as imidazoles, anilines or alkylamines.

The reported substrates are particularly valuable to medicinal chemistry, since electron-deficient systems, as well as polar but weakly basic nitrogen atoms possess molecular properties desired in biologically active molecules.

More C(sp^2^)–N bond forming reactions are reported in the literature; however, they are encountered further in this review, as they are better suited to be included in the late stage functionalisation (LSF) section.

#### 2.4 Reactions manipulating hydrocarbon backbones

The reactions of hydrocarbons are central to building the scaffolds of molecules. This is also true in medicinal chemistry. There are countless C–C bond-forming reactions using traditional chemistry and organophotoredox synthesis can offer some interesting options as well.

**Scheme 12:**
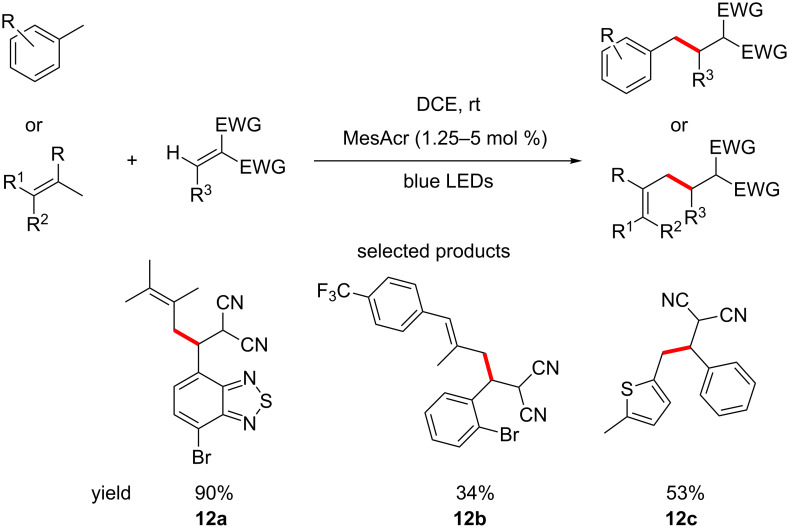
Photoredox-promoted radical Michael addition reactions of allylic or benzylic carbons.

Wu et al. reported the alkylation of unfunctionalised allylic and benzylic sp^3^ C–H bonds by reaction with Michael acceptors, using blue LEDs and acridinium salts (Scheme 12) [55]. The main advantage is the absence of strong bases like *tert*-butyllithium (*t*-BuLi).

**Figure 8:**
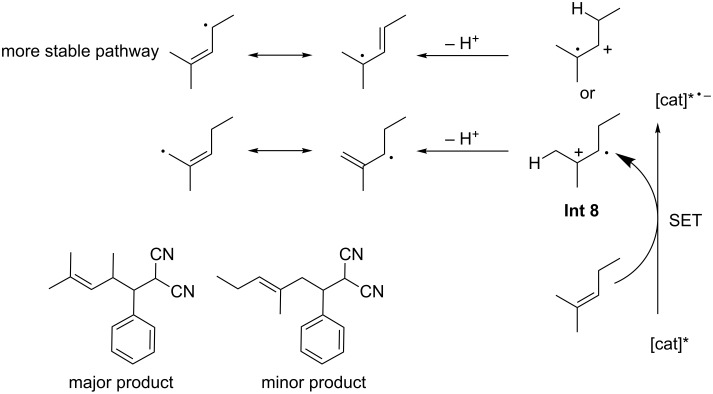
Proposed mechanistic rationale for the observed chemoselectivities.

A very broad scope of Michael acceptors, allylic and benzylic substrates is reported, with an equally broad range of yields achieved (10–99%). Some selectivity is observed when asymmetric alkenes are used. The key to this selectivity is likely the proposed intermediate **Int 8**, which is formed selectively by SET from the alkene to the excited photocatalyst, in a reductive quenching of the catalytic cycle (Figure 8).

The more stable intermediate **Int 8** is formed selectively by the SET and the allylic radical species that is formed from the less hindered and more reactive, less substituted position of its two canonical forms.

**Scheme 13:**
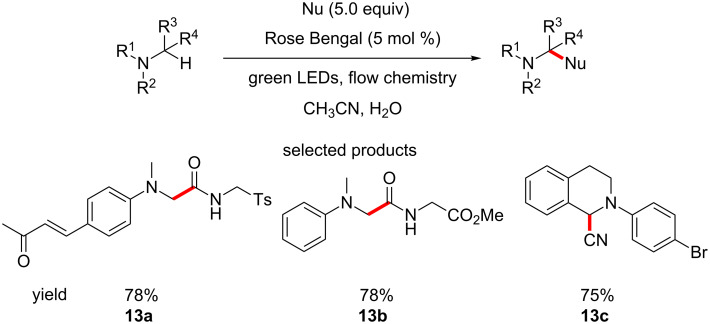
The photocatalytic manipulation of C–H bonds adjacent to amine groups.

In a similar manner, Rueping et al. demonstrated the functionalisation of C–H bonds α to tertiary amines with various nucleophiles. They also reported the formation of C–C bonds from α-amino C–H bonds using an organophotocatalytic version of the Ugi reaction. These procedures were undertaken in a flow chemistry set-up, using irradiation by green LEDs and Rose Bengal as the photocatalyst (Scheme 13) [56].

The scope of the reaction is fairly broad, especially considering the method was developed in a flow chemistry set-up, which requires a large amount of optimisation itself. A range of nucleophiles including nitroalkanes, cyanides, malonates and phosphonates are used to modify different *N*-aryltetrahydroisoquinolines. These products have the potential for quite a range of subsequent reactions for elaboration and their core structural characteristics are quite drug like.

The products of the Ugi-type reaction the group report are also interesting. The functional group (FG) compatibility of the reaction is very good, as many of the FGs tolerated can be further functionalised in a plethora of different ways. Examples of heterocyclic (e.g., pyridinyl) anilines would be more relevant to the pharmaceutical industry.

**Scheme 14:**
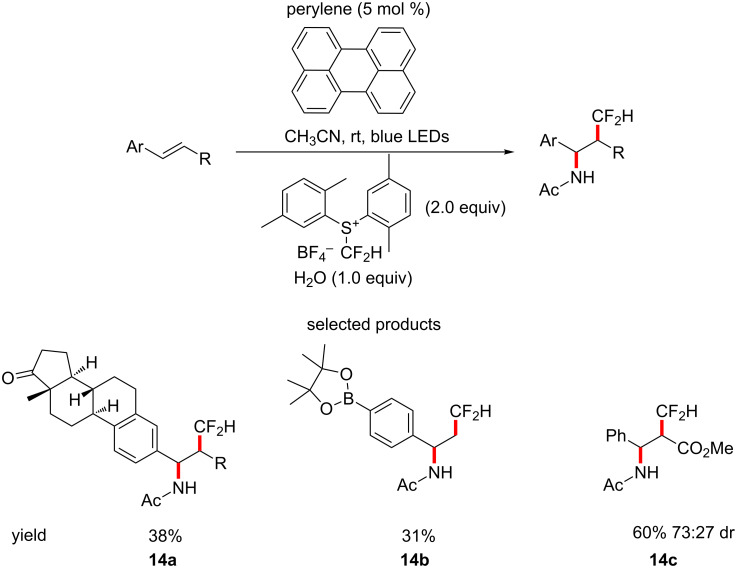
The perylene-catalysed organophotoredox tandem difluoromethylation–acetamidation of styrene-type alkenes.

Medicinal chemistry often requires particularly furnished hydrocarbon backbones as bioisosteric replacements. One such interesting group is the difluoromethyl group. Akita et al. have described a novel difluoromethylating agent which was used to simultaneously install a difluoromethyl and an acetamide group on various styrene-type derivatives, under perylene-catalysed (*E*_ox_* (cat^+•^/cat) = −2.23 V vs ferrocene in acetonitrile) photoredox conditions (Scheme 14) [57].

For the most part, the scope of the reaction is limited to relatively simple styrenes, however, some rather interesting substrates are reported, as shown in Scheme 12, in addition to some others, e.g., *meta-*aldehyde or *para-*bromo substituents. These types of motifs have the potential to be elaborated into very drug-like molecules.

Overall, this presents a decent method for the introduction of the typically difficult to introduce CF_2_H group. However, this method is applied to quite simple substrates and so use of this protocol is limited to the early steps in the synthesis of compounds. The ability to extend this procedure to encompass structurally diverse and relatively delicate scaffolds, making it suitable for LSF, would make this an incredibly valuable tool to the medicinal chemist.

### 3 Heterocycle formation

The importance and prevalence of heterocyclic systems in medicinal chemistry cannot be overstated. Although reactions for the formation of heterocycles are decreasing in frequency [45], the presence of heterocycles in drugs on the market is still extremely high. The former is more a reflection of the fact that heterocyclic building blocks are now more readily available as starting materials, so chemists opt to construct them less frequently.

**Figure 9:**
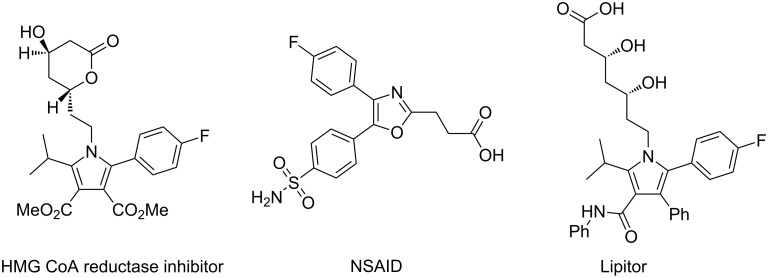
Examples of biologically active molecules containing highly functionalised five membered heterocycles.

Many biologically active synthetic compounds contain highly substituted five-membered heterocycles. In particular, pyrroles and oxazoles are quite commonly encountered (Figure 9) [58–59].

**Scheme 15:**
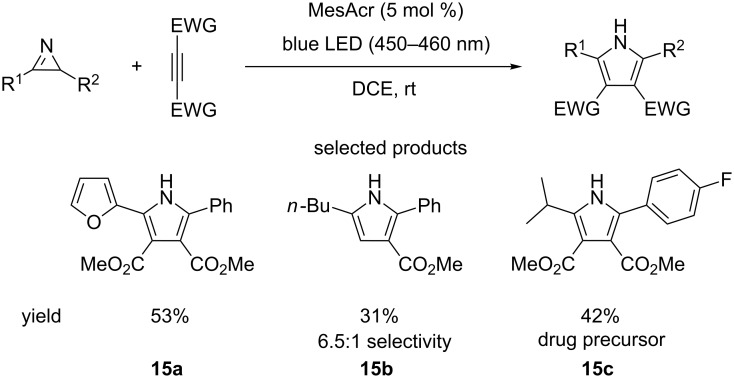
The [3 + 2]-cycloaddition leading to the formation of pyrroles, through the reaction of 2*H*-azirines and alkynes via organophotoredox catalysis.

Xiao and co-workers have shown that highly substituted pyrroles can be synthesised by the [3 + 2]-cycloaddition of electron-poor alkynes and 2*H*-azirines [60]. The reaction is performed under blue LED irradiation and using acridinium salts as a photocatalyst (Scheme 15).

**Figure 10:**
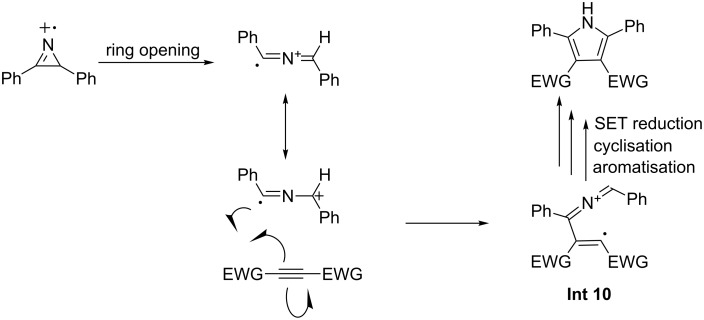
Proposed intermediate that determines the regioselectivity of the reaction.

With respect to 2*H*-azirine the scope of the reaction is demonstrated to be relatively broad, with aromatics, heteroaromatics and aliphatics all seen. However, the variation in the alkyne partner is limited, with only examples of esters and nitriles shown. The yields are variable (15–98%), with no general explanation being offered to rationalise this by the authors. An example of the reaction of two asymmetric substrates is provided and the reaction demonstrates reasonable regioselectivity (6.5:1). The synthesis of **15c**, a precursor to an active pharmaceutical ingredient (API), by the authors demonstrates how this method is immediately useful in the synthesis of biologically active molecules. Although the authors offer no direct explanation for the observed regioselectivity, the mechanism of the reaction could provide some insight. The key step of the reaction, which sets the regiochemistry of the product is seen in Figure 10.

The intermediate cation radical **Int 10** and the stability of the positive charge in the iminium radical cation are the keys in the mechanism and to understanding the selectivity of the reaction.

**Figure 11:**
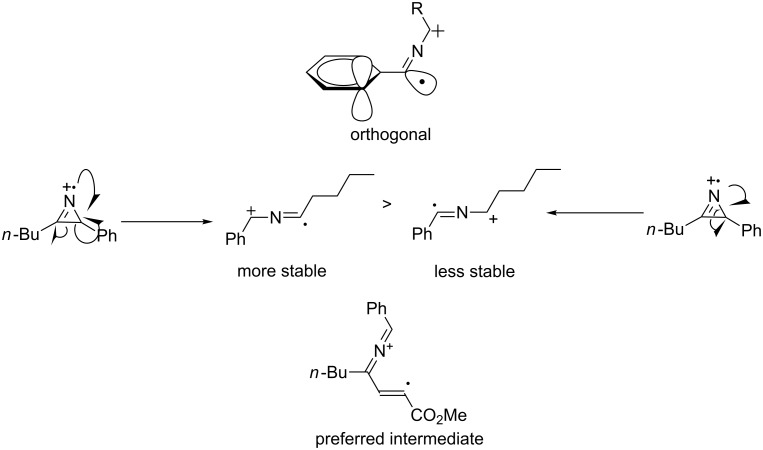
Comparison of possible pathways of reaction and various intermediates involved.

In an unsymmetrical reactant, the iminium carries most of the partial positive charge on the benzylic carbon (Figure 11). The radical is not stabilised by being borne on the benzylic carbon, as the aromatic ring must lie in conjugation with the iminium double bond, making the orbitals of the ring orthogonal to the orbital in which the unpaired electron resides.

**Scheme 16:**
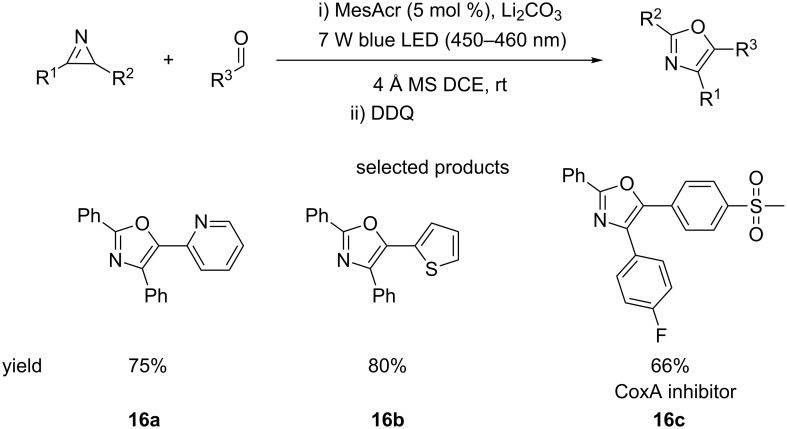
The acridinium salt-catalysed formation of oxazoles from aldehydes and 2*H*-azirines.

The Xiao group have also published a method for making oxazoles using conditions that are very similar to those described above (Scheme 16). The reaction uses 2*H-*azirines and aldehydes to access the functionalised heterocycles [61].

Unlike the pyrrole-forming reaction, this protocol requires an oxidising agent, DDQ, for the desired oxazole to be obtained. This means that access to the corresponding 2,5-oxazolines is also possible. Aliphatic and heteroaromatic substituents on the 2*H-*azirine were not tolerated. The aldehyde substituents are much more diverse, with a variety of substituted benzenes, heteroaromatics, carbonyls and aliphatic side chains undergoing the cycloaddition.

Overall, these two methods provide much more mild, scalable and environmentally friendly reaction conditions than the traditional methods employed for making these highly substituted heteroaromatics.

**Scheme 17:**
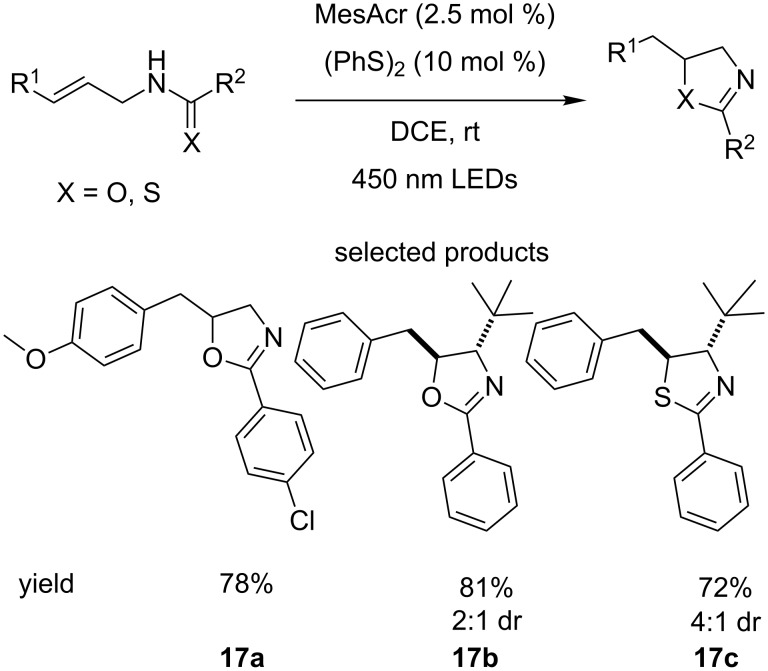
The synthesis of oxazolines and thiazolines from amides and thioamides using organocatalysed photoredox chemistry.

Access to the saturated oxazolines and thaziolines from amides and thioamides, respectively, has been described by Nicewicz who used acridinium salt photocatalysts in cooperation with a disulfide cocatalyst, which is converted to the corresponding thiol and serves as a source of hydrogen atoms for the reduction of the double bond (Scheme 17) [62].

The scope of the reaction is not particularly broad; electron-withdrawing groups such as pyridyl or trifluoromethyl are not tolerated. No functional group tolerance towards other carbonyls such as esters or aldehydes is demonstrated. The use of terminal or trisubstituted alkenes is limited and the reaction times are long.

However, the authors do report that the reaction is somewhat diastereoselective, favouring the *anti-*configuration in all cases. In addition, the option for oxidation to the oxazole or thiazole is always enticing as a way of easily accessing a diverse set of molecules.

**Figure 12:**
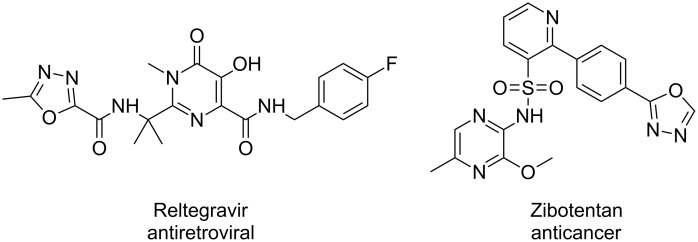
Biologically active molecules on the market containing 1,3,4-oxadiazole moieties.

Immediately akin to the oxazole moiety is the oxadiazole heterocycle, which exhibits similar properties. There are several examples of drugs on the market containing such heteroaromatics (Figure 12).

**Scheme 18:**
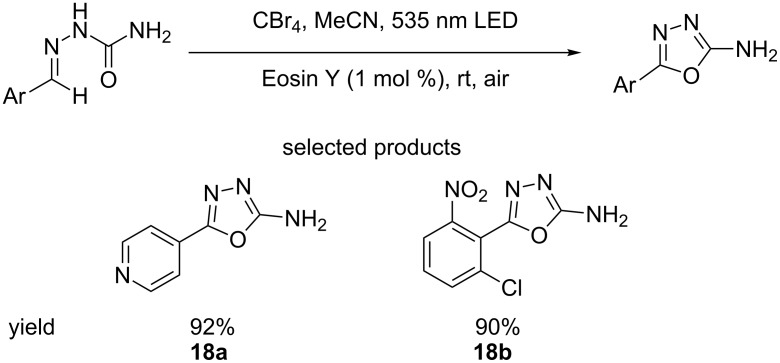
The synthesis of 1,3,4-oxadiazoles from aldehyde semicarbazones using Eosin Y organophotocatalysis.

Yadav et al. reported the synthesis of 1,3,4-oxadiazoles from aldehyde semicarbazones, using CBr_4_, green LEDs and Eosin Y as the photocatalyst, at room temperature, in the presence of air (Scheme 18) [63].

This procedure offers a much milder route to these heterocycles than traditional synthetic methodologies, which typically use harsh conditions. Semicarbazones are readily synthesised from the corresponding aldehydes, so these starting materials are easily accessible. The yields are very good to excellent (86–96%), while the variation on the ligands of the aromatic ring covers a sensible range. The 4-pyridyl **18a** example is particularly interesting, as is the hindered 2,6-disubstituted ring system **18b**. Only the synthesis of 2-amino-1,3,4-oxadiazoles is reported, which although useful building blocks, are not extremely common in drugs.

**Scheme 19:**
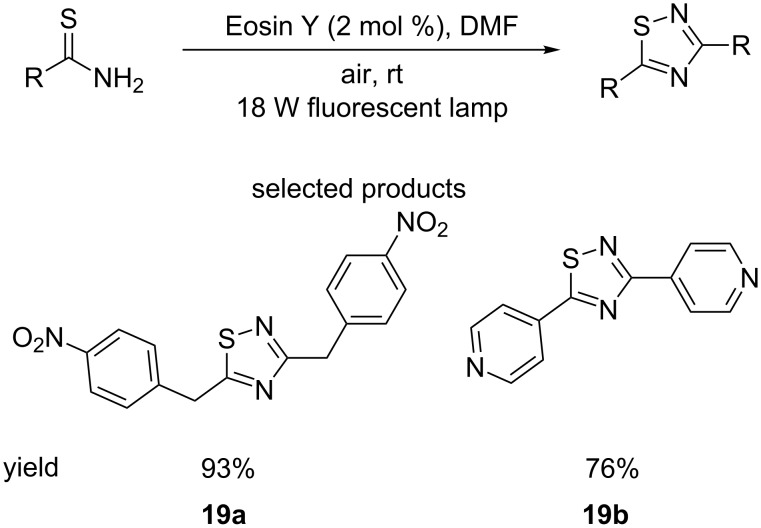
The dimerization of primary thioamides to 1,2,4-thiadiazoles catalysed by the presence of Eosin Y and visible light irradiation.

The Yadav research group have also published the homocoupling of primary thioamides for the formation of symmetrical 1,2,4-thiadiazoles, using visible light and Eosin Y as the photocatalyst, in air at room temperature (Scheme 19) [64].

The immediately obvious limitation of this reaction is the identical nature of the substituents on the product. This severely limits its potential applications, and the usefulness of this protocol is likely limited to the creation of linkers or pendant groups.

**Scheme 20:**
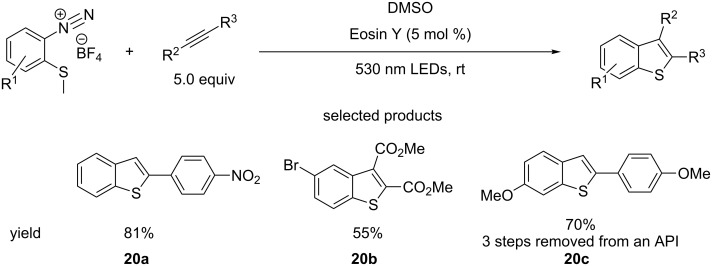
The radical cycloaddition of *o*-methylthioarenediazonium salts and substituted alkynes towards the formation of benzothiophenes.

Benzo-fused five-membered heterocycles also find widespread use in medicinal chemistry, with indoles, benzothiophenes and benzimidazoles seen in many drugs. In another demonstration of the value of diazonium salts, the König group have published a protocol for the synthesis of substituted benzothiophenes using Eosin Y photocatalysis, starting from *o*-methylthioarenediazonium salts and substituted alkynes (Scheme 20) [65].

The scope of the substrates demonstrated is quite broad, with substituted aromatic and aliphatic alkynes being used and a variety of substituents tolerated on the diazonium salt starting material. Terminal alkynes selectively formed 2-substituted benzothiophenes, whereas the regioselectivity of unsymmetrical disubstituted alkynes was not explored. It is important to note the role of DMSO, which acts as a demethylating agent as well as solvent. Even so, the benzothiophene cores constructed by the authors are still valuable, as molecules such as **20b** can be further elaborated in many ways and **20c** is a precursor to an API.

**Scheme 21:**
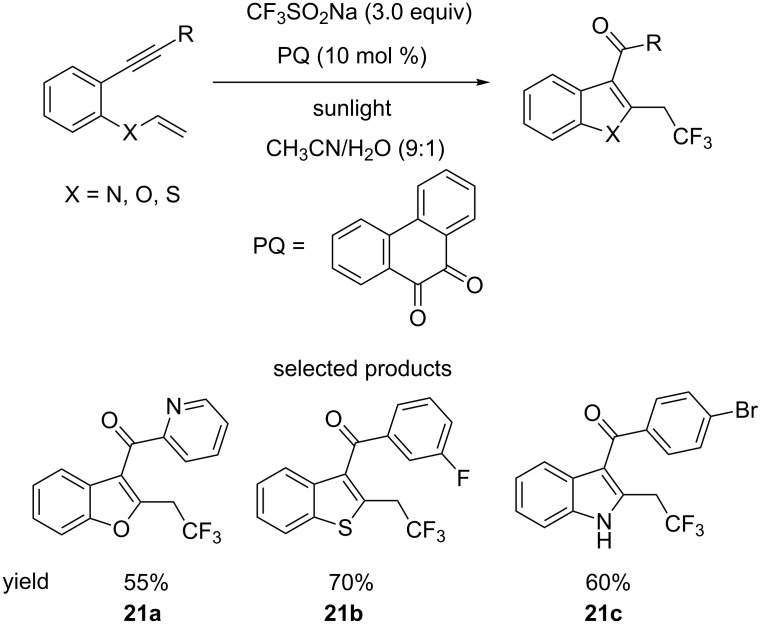
The dehydrogenative cascade reaction for the synthesis of 5,6-benzofused heterocyclic systems.

Another method for benzothiopene construction is seen in Kumar and co-workers’ report describing a dehydrogenative oxytrifluoromethylation cascade reaction of 1,6-enynes, catalysed by phenanthrene-9,10-dione (PQ) (*E*_red_*(cat/cat^•−^) +1.6 V vs SCE) using visible light (Scheme 21) [66]. However, benzofurans and, most importantly, indoles are also accessible through this cascade.

**Figure 13:**
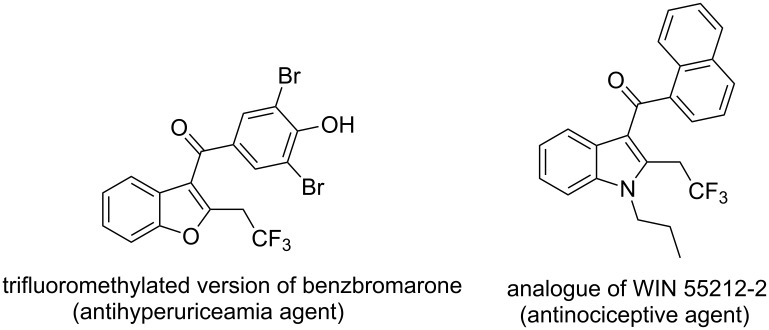
Trifluoromethylated version of compounds which have known biological activities.

The authors demonstrated the synthesis of an array of different, potentially drug-like compounds. The authors also showed the accessibility of their starting materials by synthesising the 1,6-enynes from the corresponding 2-halogenated phenols, benzenethiols or anilines, via a simple substitution–elimination–Sonogashira synthetic sequence. In addition, the group has synthesised trifluoromethylated modified versions of certain drugs (Figure 13).

**Scheme 22:**
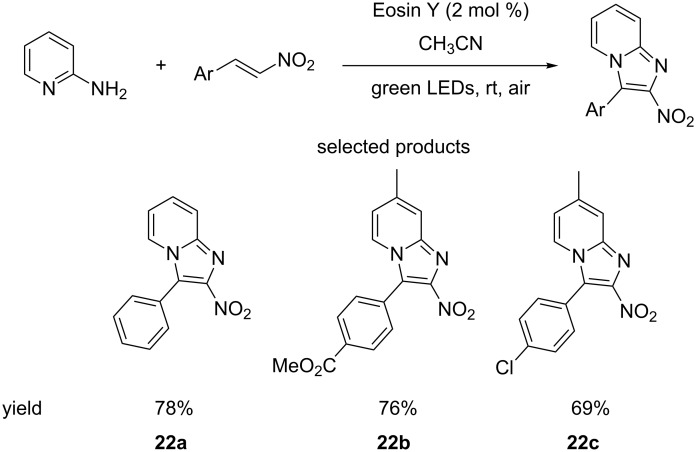
Eosin Y-catalysed photoredox formation of 3-substituted benzimidazoles.

Variations on the classical benzofused heterocycles (indole etc*.*) – such as benzimidazoles or tetrazolopyridines are often seen in medicinal chemistry. Singh et al. reported a method for preparing 3-arylnitrobenzimidazoles from 2-aminopyridines and nitroalkanes, using green LED Eosin Y photocatalysis, with molecular oxygen as the oxidant (Scheme 22) [67].

Heterocycles in drugs are not only restricted to five-membered rings. Pyridines, pyrazines, pyrimidines, pyridazines are all common functional groups in biologically active compounds.

**Scheme 23:**
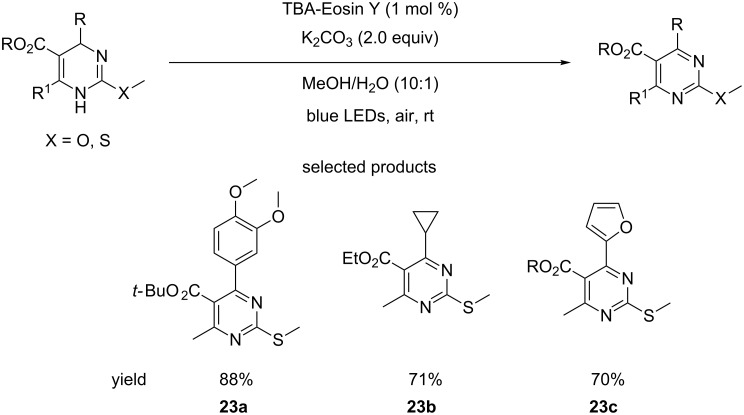
Oxidation of dihydropyrimidines by atmospheric oxygen using photoredox catalysis.

Liu et al. have published the visible light-catalysed oxidation of dihydropyrimidines (DHPMs) using atmospheric oxygen as the stoichiometric oxidant, TBA-Eosin Y photocatalysis and carbonate as the base at room temperature (Scheme 23) [68].

The group also reported that this transformation is possible using sunlight as the source of photons, with a yield comparable to that obtained when blue LEDs were employed. Though the scope of the reaction is quite broad, variation is only investigated in the C4 and C5 positions of the DHPMs. Variability at C1 and C6 are not investigated. The variation of the ester and the C4 ligands, however, is good, with some interesting products being presented.

The authors do not justify the selection of the particular substitution pattern with the C6 methyl and the C2 heteroatom methyl, e.g., ease of access to this type of DHPM core. Therefore, it would be interesting to see whether this method is compatible with other DHPM systems.

**Scheme 24:**
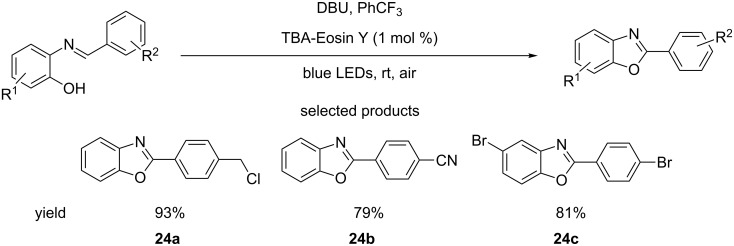
Photoredox-organocatalysed transformation of 2-substituted phenolic imines to benzoxazoles.

In the same publication, the group described the use of similar – also mild – reaction conditions to synthesise benzoxazoles from 2-substituted phenolic imines (Scheme 24). This implies that the DHPM manipulation was perhaps a preliminary study that served to optimise mild oxidation conditions.

**Scheme 25:**
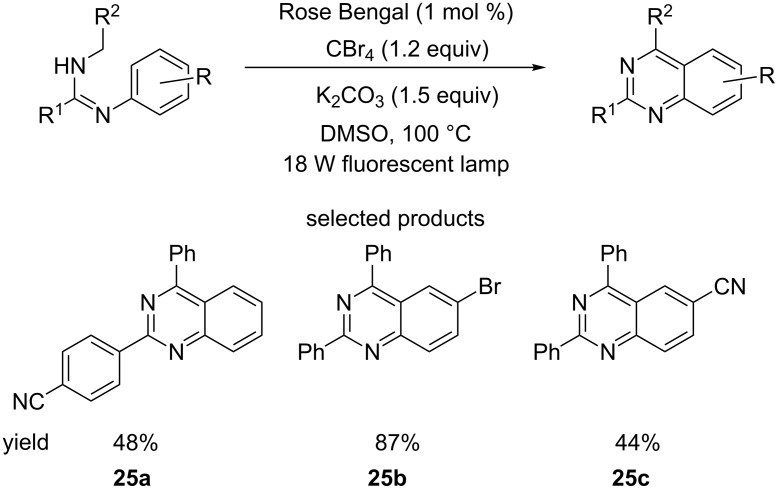
Visible light-driven oxidative annulation of arylamidines.

Tang et al. reported a procedure for performing a visible light-driven oxidative cyclisation of arylamidines using Rose Bengal as the photocatalyst, in the presence of base and CBr_4_ as the oxidant (Scheme 25) [69].

The scope of the reaction is restricted to mono-substituted benzenes and lacks any carbonyl derivatives as ligands. The reaction conditions are relatively harsh (high temperature) compared to the typical conditions encountered so far.

Overall, the synthesis of many of the most common heterocyclic systems has been reported using organophotocatalytic conditions, which offer several advantages over their traditional counterparts. Mainly, milder conditions are employed and easily elaborated structures are accessed in one step.

### 4 Late stage functionalisation

In this section, the bonds formed during the reactions are highlighted in red, as they are not always immediately and easily identifiable.

Late stage functionalisation (LSF) is a relatively new concept. It is the name given to the synthetic strategy in medicinal chemistry where lead structures are diversified by transformation of unactivated C–H bonds. In LSF C–H bonds are treated as distinct functional groups. This approach allows for diversification of lead structures without having to devise alternative syntheses [70].

There are numerous examples of novel methodologies for LSF published in recent years. The general theme is that these protocols employ mild conditions that are widely functional group-tolerant, as they usually operate on highly elaborate structures.

LSF can either be guided, e.g., selective fluorination of a molecule or can also follow an unselective approach, e.g., fluorination in various positions, but in either case the goal is exploration of SAR directly on a lead structure and easy diversification. In addition, LSF can explore the addition of small groups, e.g., methyl, fluoro, chloro, trifluoromethyl etc., or can be aimed at installing larger functional groups, e.g., heterocycles, amides or long alkyl chains.

**Scheme 26:**
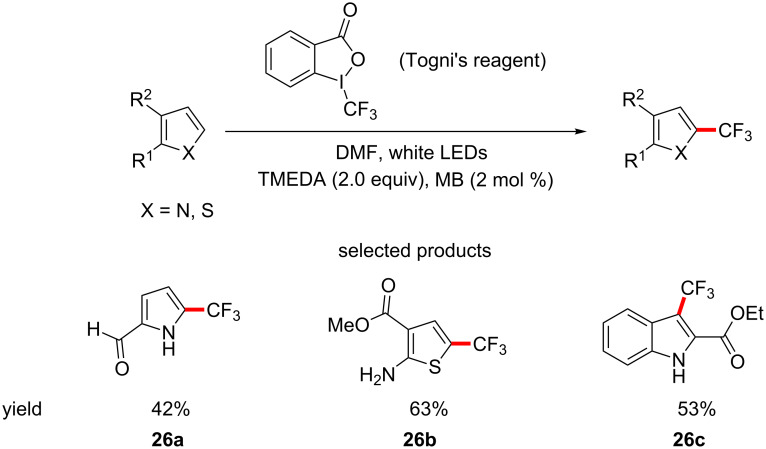
Methylene blue-photocatalysed direct C–H trifluoromethylation of heterocycles.

The previous two sections outlined how mild the reaction conditions employed in visible light organophotoredox catalysis usually are, making it a uniquely suited method for LSF. For example, Scaiano et al. have demonstrated the direct C–H trifluoromethylation of heterocycles using TMEDA, visible light from white LEDs, Methylene Blue as the photocatalyst and Togni’s reagent as the trifluoromethyl source (Scheme 26) [71].

**Scheme 27:**
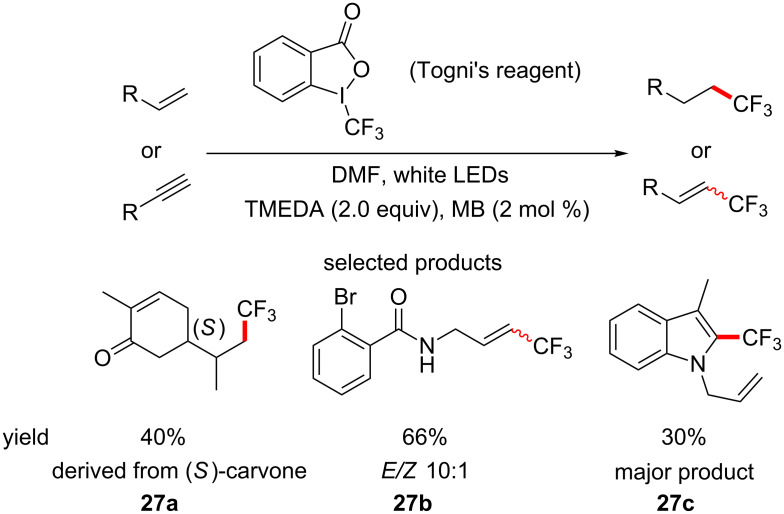
Photoredox hydrotrifluoromethylation of terminal alkenes and alkynes.

The reaction is regioselective and follows the same substitution pattern as the electrophilic substitution of electron-rich heterocycles. Although highly elaborated structures are not presented, the mild reaction conditions and general functional group compatibility that the reaction exhibits make it well suited for LSF purposes. In the same study, the hydrotrifluoromethylation of terminal alkenes and alkynes is also reported and in this case the amine base acts as the hydrogen atom source to complete the reduction of the π-bond (Scheme 27).

The more electron-rich double bond is more reactive towards the electrophilic trifluoromethyl radical, providing some selectivity to the process, which indicates that it could be applied to a guided LSF strategy.

**Scheme 28:**
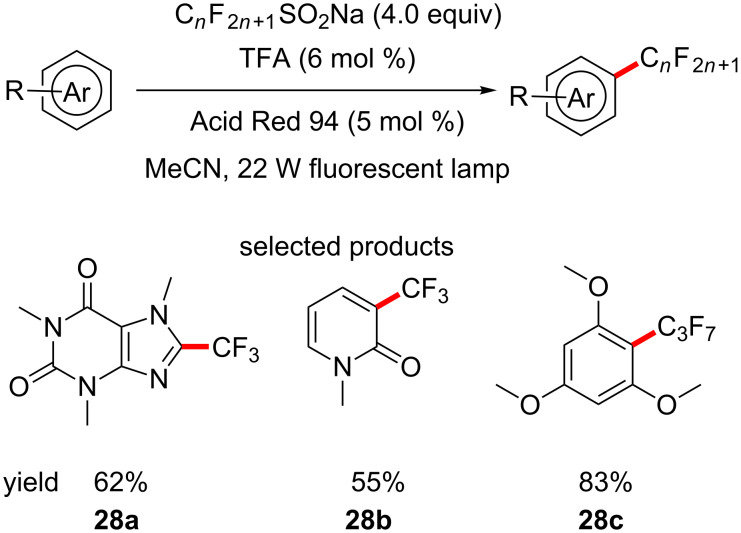
Trifluoromethylation and perfluoroalkylation of aromatics and heteroaromatics.

Itoh and co-workers have described a procedure for the direct C–H perfluoroalkylation of substrates, which can act as fluorous tags. The group utilised the corresponding fluoroalkyl sulfinate salt as the fluoroalkyl source, in combination with TFA and Acid Red 94 as the photocatalyst, under 22 W fluorescent lamp irradiation to perform the transformation (Scheme 28) [72].

The substrate scope is limited to rather simple compounds, the most structurally complex of which is caffeine. The yields are good (44–92%), with regioselectivity being observed in a few cases. As with the trifluoromethylation by Pitre et al., this procedure does fit all the criteria for LSF, as it is very mild and simple, even though no complex structures are exemplified.

**Scheme 29:**
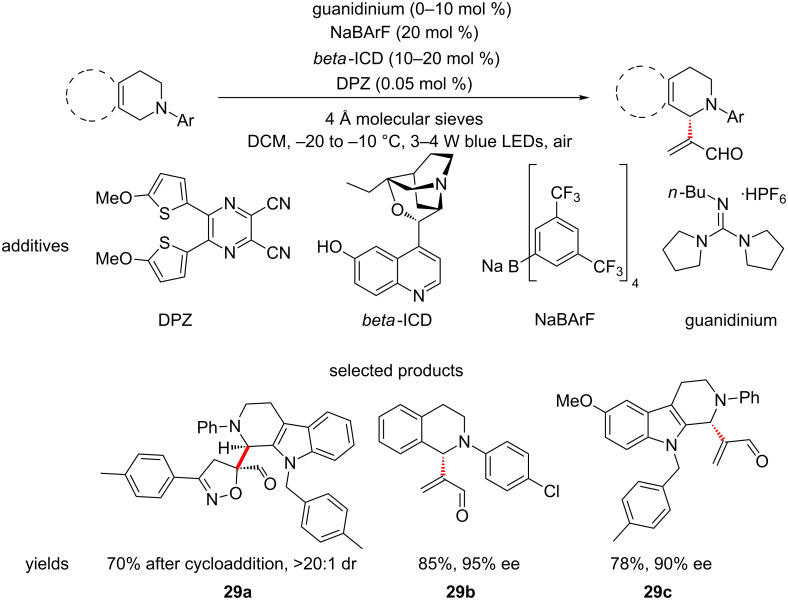
The cooperative asymmetric and photoredox catalysis towards the functionalisation of α-amino sp^3^ C–H bonds with electron-deficient olefins.

Although it does not fit the exact definition of a reaction employed in LSF, Jiang and co-workers have described a procedure that allows the enantioselective aerobic olefination of α-amino sp^3^ C–H bonds, using cooperative asymmetric and organocatalysed photoredox catalysis (Scheme 29) [73].

This may not allow for direct diversification of leads, however, the products shown can, in one or two steps, be converted into functionalised versions of a lead compound (vide infra). The study revolves around two types of substrates, tetrahydroisoquinolines (THIQs) and tetrahydro-β-carbolines (THCs), both of which are scaffolds encountered in biologically active molecules.

This reaction was included due to the ability to introduce chirality into lead structures, something that is valuable in medicinal chemistry. The straightforward synthesis of isoxazoline **29a** from the corresponding vinyl aldehyde is a perfect example of the potential for application to LSF.

**Scheme 30:**
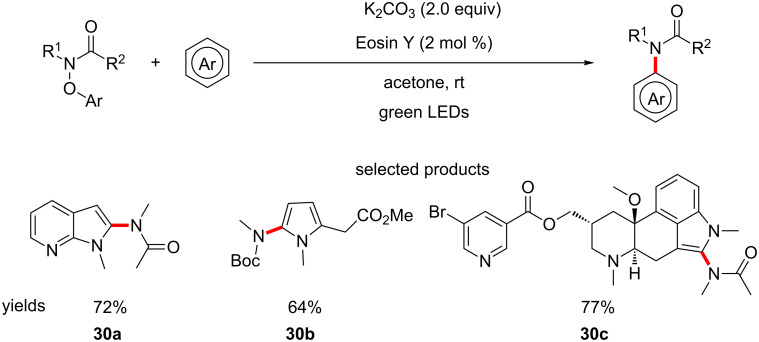
Organophotoredox-catalysed direct C–H amidation of aromatics.

Leonori et al. have demonstrated the coupling of amide derivatives to aromatics using aryloxy amides, under Eosin Y photocatalysis with green LEDs and potassium carbonate (Scheme 30) [74].

The reaction is well suited for LSF, as is demonstrated by the authors in the diversification of derivatives of lysergic acid such as **30c**. The overall scope of the reaction is quite diverse with respect to both the amide and aromatic coupling partner. The authors also address the issue of availability of the starting aryloxy amides by accessing their starting materials in two simple steps.

**Scheme 31:**
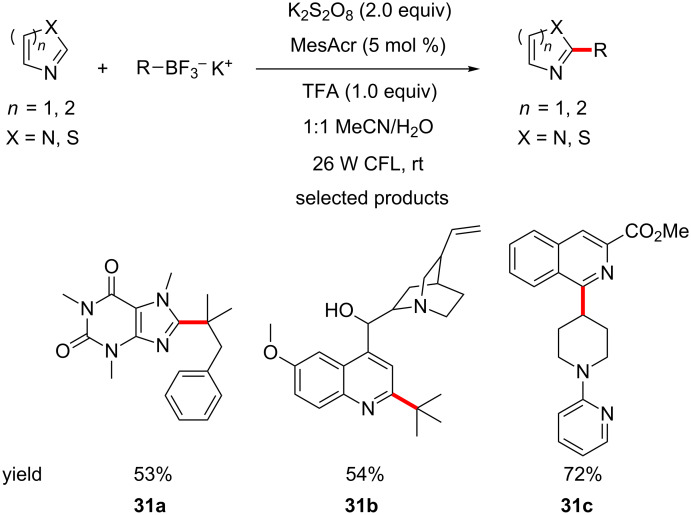
Direct C–H alkylation of heterocycles using BF_3_K salts. CFL – compact fluorescent lamp.

Molander and his group report the selective, direct C–H alkylation of various heterocycles, using their staple BF_3_K salts, visible light, persulfate and acridinium salts as the photocatalyst (Scheme 31) [75].

The scope of the reaction is truly exceptional, with a wide variety of heterocycles, ranging from nicotinamides to highly functionalised quinolines, such as the antimalarial drug quinine. The ability of this protocol to tolerate such highly functionalised molecules, with such a variety of functional groups present, really justifies the claims of the authors that this protocol is ideal for LSF in drug discovery programs.

The scope of the alkyl chains is also tremendous, with primary, secondary and tertiary alkyl trifluoroborates being used. The team also addresses the availability of these substrates, showing how these can be easily made in one step from the corresponding alkyl bromides, using a method published by Cook [76].

**Figure 14:**
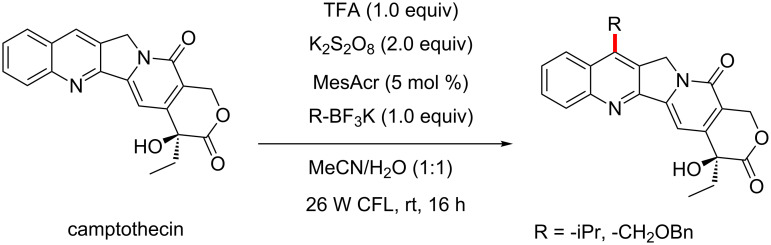
The modification of camptothecin, demonstrating the use of the Molander protocol in LSF.

The authors also further establish the immediate value of the procedure to LSF by exploring SAR of camptothecin, a molecule identified as an anticancer drug candidate. The authors selectively manipulated the C-7 position, which has been shown to improve efficacy when alkylated (Figure 14) [77].

**Scheme 32:**
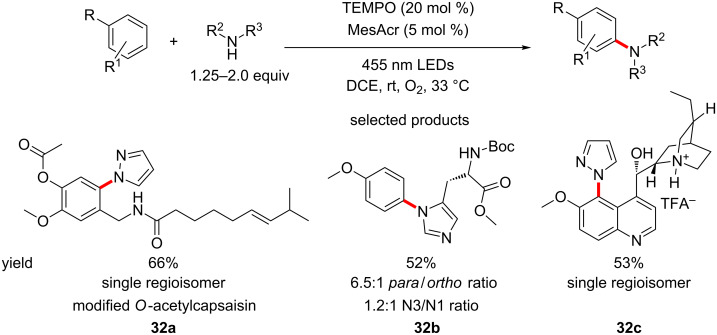
Direct C–H amination of aromatics using acridinium salts.

Nicewicz and co-workers have published a procedure for the aerobic C–H amination of aromatics, using acridinium salts as the photocatalyst under blue LED irradiation (Scheme 32) [78].

The authors have demonstrated a truly extensive scope for their protocol, subjecting a range of aromatics, heteroaromatics and fused aromatic and heteroaromatic systems with a variety of substituents to C–H amination using a wide range of heterocyclic amines. The functionalisation of molecules that are natural product-like such as **32c** is demonstrated by the authors, which is an excellent example of how this protocol translates seamlessly to drug discovery in the LSF strategy.

**Scheme 33:**
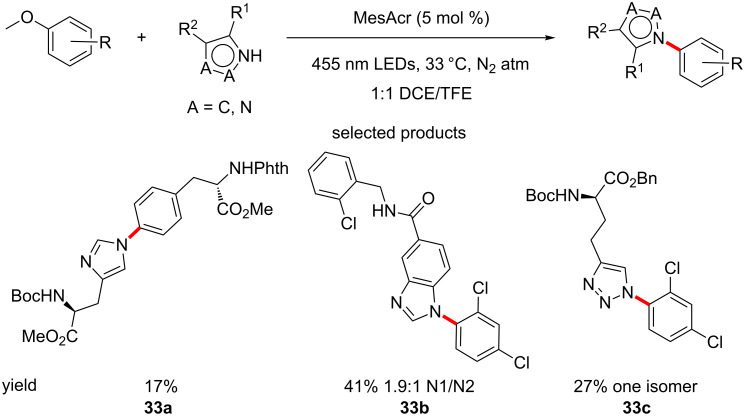
Photoredox-catalysed nucleophilic aromatic substitution of nucleophiles onto methoxybenzene derivatives.

In a method that is complementary to their C–H amination strategy, Nicewicz et al. have reported the S_N_Ar-type addition of nucleophiles to methoxybenzene derivatives at the *ipso* position, as opposed to the C–H amination that operates on the *ortho-* and *para-*position of such EDGs. The reaction is catalysed by acridinium salts under anaerobic conditions and irradiation by blue LEDs (Scheme 33) [79].

The scope of substrates able to undergo the transformation is quite broad and many, if not all the substrates that the group report are scaffolds and moieties seen in typical medicinal chemistry syntheses. There are numerous examples of amino acid-derived substrates, either as the methoxybenzene electrophile (tyrosine type derivatives) or as the nucleophile (histidine and related structures such as the depicted triazole **33c**).

Examples such as the synthesis of a modified structure of naproxen, starting from the methyl ester of the well-known NSAID, demonstrate the full power of the protocol for its use as a LSF tool. The mild conditions, selectivity on certain substrates and the great opportunity for diversification of substrates make this an ideal method for introducing nucleophilic ligands onto aromatic rings.

**Scheme 34:**
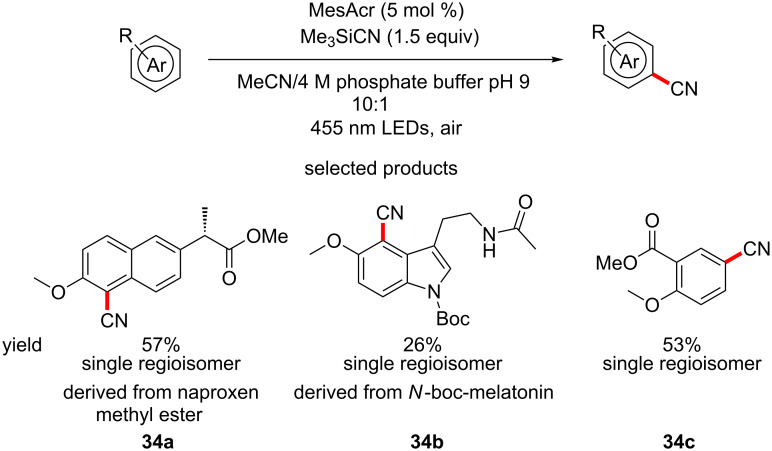
The direct C–H cyanation of aromatics with a focus on its use for LSF.

Another publication from the Nicewicz group demonstrates the C–H direct cyanation of a variety of aromatic and heteroaromatic substrates. TMSCN is employed as the cyanide source, acridinium salts as the photocatalyst, under irradiation from blue LEDs and aerobic conditions (Scheme 34) [80]. The authors show a set of diverse molecules that underwent the transformation cleanly. The group again demonstrated the LSF applications by cyanating the methyl ester naproxen derivative **34a**.

In summary, organophotoredox chemistry has been developed to be applied to medicinal chemistry in the context of LSF and appears to be very broadly applicable and robust. As LSF becomes more popular in the ensuing decades, procedures such as the ones outlined above will become both more numerous and powerful.

## Conclusion

Overall, the presented literature demonstrates that the recent developments in organophotoredox catalysis are increasingly in line with the demands of medicinal chemistry. Not only has it been shown to be highly sustainable, versatile and mild, but in some cases, it enables transformations that are notoriously challenging, cf. heteroaryl–heteroaryl coupling (vide supra).

Possibly the most attractive application of these methods is LSF. Medicinal chemists are constantly exploring SAR and LSF is the concept that will expedite this process. Protocols that can be used as tools for LSF are being rapidly developed and organophotoredox catalysis is at an advantage when compared to other approaches, due to its mild nature, as has been highlighted repeatedly.

Great advances are constantly being made in this emerging field and even so, there are still numerous possibilities to be explored. For example, stereoselective photoredox chemistry is still quite sparse in the literature. Late-stage fluorination protocols are also rare and would be exhaustively used by the pharmaceutical industry. In addition, as has been pointed out in this review, mostly electron-rich heterocycles are manipulated, which are less valuable to the drug discovery process than their electron-poor counterparts. The growing number of academic and pharma laboratories entering organophotoredox catalysis and the development of even stronger photocatalysts ensures that the field will produce impactful research for years to come.

## Abbreviations

**Table 2:** Abbreviations.

abbreviation	explanation

API	active pharmaceutical ingredient
BOC	*tert-*butyloxycarbonyl
DCC	dicyclohexyl carbodiimide
DDQ	2,3-dichloro-5,6-dicyano-1,4-benzoquinone
DMP	Dess–Martin periodinane
DMSO	dimethyl sulfoxide
EDG	electron-donating group
ET	electron transfer
EWG	electron-withdrawing group
GABA	γ-aminobutyric acid
HATU	1-[bis(dimethylamino)methylene]-1*H*-1,2,3- triazolo[4,5-b]pyridinium 3-oxide hexafluorophosphate
IBX	2-iodoxybenzoic acid
LED	light-emitting diode
LSF	late stage functionalisation
MB	methylene blue
MesAcr	mesityl acridinium salt
NSAID	non-steroidal anti-inflammatory drug
PET	photoinduced electron transfer
SAR	structure–activity relationship
SCE	saturated calomel electrode
SET	single electron transfer
TFA	trifluoroacetic acid
TMEDA	*N,N,N′,N′*-tetramethylethane-1,2-diamine
TMSCN	trimethylsilyl cyanide
UV	ultraviolet
X	heteroatom
